# The experiences of clinical psychologists in treating traumatic stress at a tertiary psychiatric hospital in the Eastern Cape: A qualitative study

**DOI:** 10.4102/sajpsychiatry.v28i0.1868

**Published:** 2022-08-01

**Authors:** Kuriesha Munishvaran, Duane D. Booysen

**Affiliations:** 1 Department of Psychology, Faculty of Humanities, Rhodes University, Grahamstown, South Africa

**Keywords:** clinical psychologists, psychiatric hospital, patients, traumatic stress, vicarious trauma, secondary trauma, trauma narratives, vicarious post-traumatic growth; South Africa

## Abstract

**Background:**

Qualitative data on the experiences of treating post-traumatic stress disorder (PTSD) in a psychiatric setting in a low-resource context is sparse.

**Aim:**

The authors aimed to explore the lived experiences of clinical psychologists who treat patients who are either trauma survivors or perpetrators in a psychiatric hospital.

**Setting:**

A public psychiatric hospital in the Eastern Cape, South Africa.

**Method:**

A total of six individual semi-structured interviews were conducted with three clinical psychologists. Data were analysed using interpretive phenomenological analysis (IPA).

**Results:**

The analysis for each participant identified several significant themes, namely (1) support as a male person, (2) being appreciative, (3) difficult trauma narratives, (4) a mother and a psychologist and (5) fear and hopelessness.

**Conclusion:**

Treating traumatic stress amongst clinical psychologists working in a public psychiatric hospital can lead to experiences of vicarious trauma and traumatic stress. In addition, the participants experienced an added danger in treating high-risk state patients, exposing psychologists to traumatic stress. Furthermore, psychologists recognised the influence of gender and race and its impact on their roles in their personal and professional lives as practitioners treating traumatic stress.

## Introduction

Post-traumatic stress disorder (PTSD) is a global mental health priority, especially in low- and middle-income countries such as South Africa.^1,2^ In South Africa, 73.8% of the general population are exposed to at least one traumatic event, and more than 50% of South Africans experience multiple traumatic events.^3,4,5^ To this end, South African society experiences some of the most violent crimes and trauma-related experiences in the world.^6^

Several psychological treatments for PTSD have been developed over the last four decades.^7^ Yet health professionals such as clinical psychologists are still at risk psychologically when treating persons with PTSD.^1,8^ Treating PTSD is understood to be demanding as it requires recounting of the trauma narrative using various psychotherapies, such as cognitive behavioural therapies.^9^ However, listening to the details of patients’ trauma narratives exposes clinicians to traumatic stress, vicarious trauma and secondary trauma.^8,10^

In the United States of America, between 40% and 85% of health and service practitioners develop vicarious trauma and secondary trauma, leading to burnout and compassion fatigue.^11^ Vicarious trauma is an impaired view of the self, others or the world. This occurs in response to repeated exposure to trauma narratives when a mental health practitioner engages empathically with traumatised clients.^12^ Secondary traumatic stress occurs when mental health practitioners are exposed to patients’ traumatic experiences and develop symptoms of traumatic stress themselves.^11^ Symptoms include somatic responses, negative emotions and psychological distress.^13,14^

A study based in the United Kingdom, including 253 mental health practitioners treating trauma clients, found that 70% of the scores indicated a high risk of secondary trauma.^14^ Concurrently, research in South Africa found secondary trauma to be experienced by mental health practitioners and emphasised the need to provide training for these practitioners, thus enabling them to cope with the emotional impact of treating trauma clients.^15^

In contrast, individuals who treat trauma also develop a positive belief system that leads to psychological growth and resilience.^15,16^ Positive findings in mental health practitioners treating trauma include experiences of vicarious post-traumatic growth in their personal lives.^8,10^ Vicarious post-traumatic growth is the development of positive emotional, cognitive and spiritual changes in response to direct exposure to trauma survivors’ narratives. It is associated with life satisfaction, psychological and physical wellbeing.^13^

Qualitative research is sparse on experiences of clinical psychologists treating PTSD, specifically in inpatient public service psychiatric hospitals. Public psychiatric hospitals and private hospitals differ in terms of resources, challenges and case load.^17^ For example, and contrary to the higher resourced private sector, public mental health facilities only receive an estimated 5% of state financial investment, which is even lower at provincial levels in South Africa (2.1% – 7.7%).^17^ A range of studies have investigated the impact on mental health practitioners who treat trauma survivors in communities and private practice. However, there still remains an unexplored interest in the phenomenological experiences and challenges faced by clinical psychologists working with trauma narratives in psychiatric settings. Expanding on the literature in this study is distinctive because it provides qualitative insights into the phenomenological experiences of clinical psychologists who work with trauma survivors, specifically in a public psychiatric hospital. To this end, this study was guided by the following research question: how do clinical psychologists experience working with traumatic stress in an inpatient public psychiatric hospital?

## Research methods and design

A heterogeneous purposive sample was recruited, which is recommended by interpretive phenomenological analysis (IPA).^18^ Four public psychiatric hospitals in the Eastern Cape and Western Cape were approached to participate in the study, of which only one site had interested participants. Notably, because of the small number of clinical psychologists in psychiatric facilities, some prospective participants were concerned that they would be identified and opted to not participate. A sample of three voluntary participants with the age range of 40–53 years were recruited from a psychiatric hospital in the Eastern Cape. All participants were clinical psychologists who are registered with the Health Professions Council of South Africa (HPCSA) and had work experience with varied psychopathological cases, including trauma.

Interpretive phenomenological analysis studies are known to use small idiographic samples for a deeper, detailed understanding of participant’s perceptions.^18^ The inclusion criteria were based on levels of work experience. Participants who had more than three months of work experience as a clinical psychologist at the psychiatric hospital were invited to participate in the study.^18^ The current participants had an average of 18 years of work experience in a public psychiatric hospital.

### Data collection

Interviews were between 60 and 90 min, in person, at the psychiatric hospital. In total, six interviews were conducted by K.M. in a manner which was open-ended. Three of these interviews were follow-up interviews one week after the first interview to facilitate a reflective account of participant experiences. After the initial interviews, K.M. and D.D.B. reflected on the initial interviews and identified relevant areas to further explore and clarify in the follow-up interviews. Moreover, follow-up interviews allowed for more accurate data collection and analysis, which helped us and the participants to refine certain questions, thoughts and reflections. It also aided in uncovering the potential layers of their professional responses and getting to a point of phenomenological understanding.^19^ Interviews were conducted using an audio-recorder, with the participants’ signed consent.^18^ All data were transcribed using a transcriber who was a graduate student and who also signed a confidentiality agreement.

### Interview guide

An interview schedule was developed and underwent refinement to align with the aim of the study.^18^ Items for the first interview focused on areas of interest. Item areas with probing questions included how they came to work in a psychiatric hospital and their experiences working there. The participants’ opinions and experiences of treating trauma and PTSD were also explored, along with their coping strategies. The follow-up interview included questions based on the accounts of participants’ experiences in the first interview, clarifying their experiences and obtaining deeper reflections of working with trauma victims. Treating trauma based on their gender came up as an area of interest in the subsequent interviews.

### Data analysis

This analysis took a double hermeneutic and idiographic approach by making sense of participants’ experiences in a given context, whilst also interpreting and contextualising these individual experiences. The data retrieved were analysed using the IPA analysis guidelines – in other words, a step-by-step analysis.^18^

The process first involved reading and rereading each transcript to create an overall impression for each participant. The transcript was placed in a single column with two subsequent columns for notes and subthemes. Comments and notes were made on significant reflections in the second column, and broad subthemes were identified based on these notes in the third column for each transcript.

The analysis deviated slightly as transcripts were then reread and the subthemes were numbered according to the page numbers of the participants’ narratives. Two new columns were made in a separate document, clustering new superordinate themes with subthemes according to similar patterns. Subthemes were reviewed and refined thereafter. Thoughts and reflections of the interviews were noted down and kept in a journal by K.M., which was later considered whilst refining, selecting and writing up the overall findings. Lastly, data analysis and writing of the results underwent an iterative process between both authors discussing and reflecting on the analysis of the data.

### Ethical considerations

Ethical clearance was granted by the Rhodes University Ethical Standards Committee (reference number: 2020-1341-3524), including permission from gatekeepers at the Psychology Department of the psychiatric hospital. Permission was also granted by the Department of Health (reference number: EC_202011_013). Interested participants received the consent form and interview schedule with interview questions prior to the interviews. Each participant consented verbally and signed a consent form.

## Results and discussion

Table 1 presents a list of all the themes per case. Each theme elicited from the data describes unique aspects of the individual’s professional and personal experiences working with traumatic stress within a psychiatric hospital. The themes for each participant are individually presented to maintain the idiographic experience of each participant. We used pseudonyms to protect the identities of the participants.

**TABLE 1:** List of super-ordinate themes.

Participants	Themes
Allen	Support as a male: ‘apologising for the fact that I’m it’
	Being appreciative: ‘it makes me appreciate it more and my history’
Annie	Difficult trauma narratives: ‘how do I know this about this person and do my job to help him?’
	A mother and a psychologist: ‘every time I’ll see a victim, I’m gonna see my kids’
Ruby	Fear and hopelessness: ‘I need to always be on guard’

### Participant 1: ‘Allen’

For Allen, two primary themes were identified, namely support as a male person and being appreciative.

#### Theme 1.1 Support as a male person

Allen emphasised a prominent theme across his narratives that is associated with his role as a male clinical psychologist treating traumatic stress. He acknowledged that many of his trauma patients are female, most of whom have been sexually assaulted or raped by a man; this evokes an uncomfortable awareness in the therapeutic relationship:

‘So I was in front of a lot of young coloured^1^ girls from 17 and going up into the 30s and 40s who’d been raped by a man, and here they are sitting in front of a man and there not being anybody else. And first of all, needing to talk about that and on some level, apologising for the fact that I’m it. The sad thing is, for many of them that was too much, and there’d be some kind of inauthentic goodbye at the end of the first session and they wouldn’t return.’ (‘Allen’, male, clinical psychologist)

Allen referred to a sense of helplessness experienced by both himself and the patients. Perhaps he felt that they were stuck with him in the room as their therapist, alluding to a process they felt somewhat forced into, where ‘choice’ was denied them. For Allen it could be that there was an aspect of each woman’s trauma narrative that was linked to him being male; thus, he referred to himself in an emotionless manner as ‘it’. The unspoken ‘it’ was understood to be the uncomfortable experience in the room, unsure of how it should be addressed as it created an uncomfortable encounter, knowing that being male added to the complexity of connecting with his female patients. He further reflected:

‘When a man does a terrible thing like that to a woman, so many things can be misinterpreted by a male therapist – not the male therapist doing the misinterpreting, but the client misinterpreting. It’s hard. It’s very hard, because that’s not what you’re saying; you’re not saying it’s their fault.’ (‘Allen’, male, clinical psychologist)

As a male therapist, Allen was aware of the intricacies and sensitivities of supporting female rape victims as a male therapist. Allen recognised that patients have their own perceptions based on their experiences and that he needed to hold and process these somewhat distorted perceptions.

Allen also made reference to his feelings of sadness and irritability when treating trauma victims of domestic abuse. He stated, ‘It makes me sad that there are people living lives that are empty, where there’s fear in a situation like this.’ He captured a sense of the patients’ unhappiness and fears living in an ‘empty’ home with an abuser, feeling sympathy for the patient who was living a life of no self-worth. He observed that the trauma they experienced was taking everything out of them and depleting them, which made him feel sad for the victim. Yet his experiences also compelled him to make sense of the perpetrator causing such emotional turmoil, wondering about the perpetrator’s upbringing. This appears to be a strategy he developed to help him manage his own feelings of frustration with the patient’s situation in order to hold the therapeutic frame. Allen reported:

‘The problem is I can’t allow my sympathy to come into that room. So maybe in a situation like that, the battle is between sympathy and empathy, irritability towards the other and empathy; why is he this way, what was his dad like, what was his grandfather like, how does he turn into this man? That’s also sad.’ (‘Allen’, male, clinical psychologist)

In contrast to feeling sorry for the patient, Allen empathised with their perpetrator by wondering about his life challenges and how that may have contributed to him becoming the abusive person he is. As a result of the context in which he worked, he held himself back from feeling sympathy. Instead, he tried to make sense of the perpetrator through his patients’ experiences and perhaps thought that he could help the patient by understanding the perpetrator. This led to him feeling frustrated and empathetic as he chose to be concerned about the perpetrator too.

#### Theme 1.2 being appreciative

In this theme, Allen described how his experiences had made him more appreciative of his own life. Listening to the traumatic narratives of his patients, Allen formed an appreciation for his life:

‘It makes me realise that irrespective of whatever has happened to me in my life, there are other people out there who have had it much worse than I. So, actually, it doesn’t make me down on life, it makes me appreciate it more and my history.’ (‘Allen’, male, clinical psychologist)

Allen learned from his patients’ difficult life experiences and felt grateful for his life journey when comparing himself with them. This experience seemed to have formed his personal view of life, appreciating and focusing on the little things that were pleasurable. Being a man who was called to the military for national compulsory service, working in townships and other communities, he felt empathy towards others for their struggles. He had encountered and witnessed first-hand traumatic injustices being committed and, in this way, had been exposed to the darkness of the world. His current profession enabled him to make a difference, whilst valuing his life and the opportunities afforded to him.

### Participant 2: ‘Annie’

#### Theme 2.1 difficult trauma narratives

Annie described an experience of treating child rape victims, particularly cases of incest: ‘they’re more vulnerable, and it just dials up certain responses in me; a lot of anger.’ With cases of incest, this sentiment may have left her in disbelief at how a father or other male family figure could have the capability to inflict pain on a minor. Furthermore, Annie described that she read an inpatient’s file before seeing him and was dismayed by the horrific details of his criminal charges:

‘I closed the door and I wept for an hour because I was so […] maybe traumatised from reading what this person had done and was horrified by it, and then trying to consolidate how do I know this about this person and do my job to help him? And something I reflect on many times is that I don’t have that response anymore.’ (Annie, female, clinical psychologist)

Her response to the case reflected overwhelming emotions as she was distressed by the nature of the crime the patient had committed. Annie reflected on her thoughts of being able to tolerate horrific trauma narratives, referring to the fact that she was still able to do her job. She was able to distance herself from cases through the use of her own coping strategies, these being self-awareness, personal supervision and mindfulness, whilst also (in Annie’s words) learning to apply ‘acceptance and trust with the use of collegial support.’

#### Theme 2.2 A mother and a psychologist

The following theme describes Annie’s experience of being a mother and her role as a clinical psychologist. Annie described an opportunity to get involved with victim assessments and young offenders, a forensic branch of psychology she enjoyed:

‘I thought about it, and it was interesting because there was such a tension between the professional clinical part of me and the personal part of me. Every time I’ll see a victim, I’m gonna see my kids, and I don’t want that to affect how I bring them up.’ (Annie, female, clinical psychologist)

Annie appeared to be aware of the internal tension between her professional curiosity and the potential personal cost of working within a forensic space. The commonality of trauma shaped her way of thinking as she oscillated between her role as a mother and a psychologist. In response to the trauma she worked with, Annie described a sense of fear she experienced leaving her children with the nanny or around the gardener, even after interviewing them:

‘Could I trust this person on my property whilst my kids are running around? Sometimes I look at how great they interact with the kids and I base it on that.’ (Annie, female, clinical psychologist)

She grappled with issues of trust in others because of her experience of treating both victims and perpetrators. She was aware that being in her profession came at a cost, as she had developed a distorted view of society and lost faith in humanity. For her, this evolved into becoming an overprotective nurturer: ‘I’ve spoken to [my child] about stranger danger, and it kills me to shatter that naïveté that the world is a good place.’ Although she observed her children’s positive interactions with the nanny or gardener, Annie considered her children’s freedom and wanted them to think and play as children should, as opposed to worrying about the dangers of the world: ‘so decisions about the kids’ freedom is significantly thought about very carefully.’ To this end, she found a balance by trying to make calculated decisions based on risk, whilst being careful not to instil her fears within her children.

### Participant 3: ‘Ruby’

#### Theme 3.1 Fear and hopelessness

This theme describes how Ruby experienced working in settings characterised by elements of danger, fear and hopelessness. Ruby reported being used to treating and assessing highly dangerous patients; as a result, she did not see the situation as an immediate personal danger. She reported:

‘I need to always be on guard. It made me quite hypervigilant after I needed to make sure that I keep the security quite alert. So I felt like I had two jobs to do because, for some reason, most of the security guards often fell asleep, so I kept having to bring them back to life.’ (Ruby, female, clinical psychologist)

Here, a sense of fear illuminated an always-present threat. Ruby found that she was not always safe, even with a security guard. In order to protect herself and alleviate her anxiety, she managed her fears practically by keeping the security guard awake. She alluded to how she reacted afterwards: ‘only afterwards did all the “what ifs” go through my mind.’ She responded naturally to the danger, becoming anxious as she ruminated about possible scenarios of how the dangerous patient might have harmed her, similar to the narratives of her trauma victims. Her accounts linked to intense interpersonal transferences in the workplace, as she was conscious of what the inpatient was capable of doing; therefore, she moved between feeling unprotected by the guards, having to defend herself and feeling unsafe.

Furthermore, when assessing whether a child was able to testify or not, she found this to be the most difficult and recalled becoming overwhelmed with emotion in a particular case. She stated:

‘It happened at the time when I’d just had my little girl, so looking at her crying sitting up there and I see my daughter and think, “Oh, my gosh.”’ (Ruby, female, clinical psychologist)

Ruby was reminded of her daughter when she saw the little girl in court, astounded at how a little girl could go through such emotional turmoil at such a young age. Here, she was triggered by her own recent childbirth and, during the interview, reflected on her experiences as a mother and seeing the child’s helplessness first-hand. Ruby shared that when children were hurt it triggered negative emotions within her; however, she took the responsibility upon herself, internalising the little girl’s hopelessness, evidenced by the following statement: ‘so I would pick up their helplessness. I feel like I’m going to give them justice; the guy is gonna be incarcerated but is that enough?’ In her professional capacity, she believed that she could do everything to give the victim justice. However, she reported feeling stuck, unable to gift her child patients with the jewels of freedom to live without fear.

Additionally, Ruby’s exposure to trauma narratives made her hypervigilant, specifically related to her children: ‘I have kids of my own, and it made me become more overprotective and hypervigilant, to a point that I think I became neurotic.’ Her exposure distorted her perception of society and she perceived the world as more terrifying.

## Discussion

This study found that the lived experiences of clinical psychologists working with trauma in a psychiatric context is complex, overwhelming and at times an enriching experience. In the existing literature, little is known about the nature and psychological treatment of traumatic stress in psychiatric hospitals; however, the findings of this study have brought much-needed attention to the notion that treating trauma comes with both risks and benefits.

All three participants reflected on the peculiarity of working in contexts that present a certain personal risk. Olashore et al.^20^ explored the prevalence and predictors of PTSD in a psychiatric setting in Botswana, where findings depicted that mental health practitioners who were physically harmed by patients were at risk of PTSD. Furthermore, participants highlighted powerful complex meanings related to the sociopolitical context whilst treating trauma in a psychiatric hospital. This finding speaks of the overall message that trauma is not devoid of its social context of politics and gender.^1^

The experiences of the participants made reference to gender, suggesting that sociodemographic factors perpetuate challenges when treating trauma in a psychiatric hospital. Issues of gender, race and politics are not readily referred to in the treatment of trauma, yet there is an awareness of the interplay between trauma and the social context from which it stems. For example, Allen referred to his role as a white male clinical psychologist treating ‘coloured’ trauma victims in post-apartheid South Africa, reflecting on how his presence affected the therapeutic process.

The female participants reflected distinctly on family and work. Specifically, motherhood emerged prominently as Annie and Ruby’s experiences of treating trauma influenced how they parented their children and how they themselves felt about the safety of the world around them. Annie and Ruby’s experiences reflected suspicious and hypervigilant behaviours in their personal lives, which influenced their protective roles as mothers.

Vicarious symptoms of hypervigilance seem to be more common for Annie and Ruby, whilst Allen had no noticeable concerns for safety. Arguably, the difference in experiences alludes to how men and women might experience treating trauma, especially in a psychiatric hospital, in ways that are diverse in terms of gender. This finding concurs with the existing literature that women are more at risk of traumatic stress than men and treating traumatic stress does present a mental health risk to the practitioner.^21,22^

Considering the complexity of the participants’ experiences, coping and making sense of their experiences occurred at varying levels. For example, Annie highlighted the importance of personal and professional boundaries, collegial support and psychological flexibility and acceptance. Considering the emotional intensity of trauma work, clinicians should maintain a higher level of self-care to mitigate and/or prevent the onset of burnout and vicarious traumatisation.^13,23^

Lastly, the study not only explores the complexity of working in the field of traumatic stress but also alludes to peripheral variables that could add deeper levels of complexity. For example, encountering trauma narratives in a psychiatric context could be experienced as a double-edged sword, requiring enhanced psychological flexibility on the account of clinicians. Furthermore, experiences related to socio-economics, politics and gender are found within the broader context of where trauma and the treatment of traumatic stress occurs. Therefore, understanding how the broader context can influence both survivor and clinician are required when treating traumatic stress.

## Limitations of the study

This study has a major limitation, namely that it was conducted at a public psychiatric hospital, which limits our understanding of different settings in South Africa. In future, various psychiatric facilities should be included to further explore the experiences of clinical psychologists who treat traumatic stress in psychiatric hospitals.

## Contributions

Notwithstanding the methodological limitations, the study does provide insights into the peculiar context of treating traumatic stress in a psychiatric setting. The treatment of traumatic stress could arguably be an overlooked process because of the common focus on more severe psychiatric conditions. Yet the study has highlighted the pervasiveness and complexity of violence and trauma experienced by patients and professionals in a public psychiatric hospital.

## Recommendations

It is further recommended that future research focus on how sociopolitical factors and dangers in the workplace influence clinical psychologists’ service delivery. In addition, content on healthy coping strategies, vicarious trauma, boundaries in professional and personal roles, dangers in psychiatric hospitals and sociopolitical considerations could be included in training programmes to enlighten future clinicians on the implications of working with trauma in psychiatric hospitals.

## Conclusion

It is evident that challenging and perplexing experiences do arise when clinical psychologists treat trauma survivors at a psychiatric hospital, which can lead to vicarious trauma and traumatic stress. This study observed an added element of danger whilst treating high-risk state patients, thus exposing psychologists to traumatic stress. Furthermore, a sociopolitical aspect to treating trauma that was not found extensively in previous research was found in this study. Psychologists recognised their gender and race and the impact this has on their roles in their personal and professional lives whilst working within the field of trauma.

## Footnotes

1 The term ‘coloured’ was used as a racial classification category under the Population Registration Act of the apartheid dispensation and is still commonly used in South Africa today to refer to individuals who are of indigenous ethnicity (e.g. KhoeKhoe and San).
